# VEHIOT: Design and Evaluation of an IoT Architecture Based on Low-Cost Devices to Be Embedded in Production Vehicles

**DOI:** 10.3390/s18020486

**Published:** 2018-02-06

**Authors:** Jonatan Pajares Redondo, Lisardo Prieto González, Javier García Guzman, Beatriz L. Boada, Vicente Díaz

**Affiliations:** 1 Mechanical Engineering Department, Institute for Automotive Vehicle Safety (ISVA), Universidad Carlos III de Madrid, Avda. de la Universidad 30, 28911 Leganés, Madrid, Spain; bboada@ing.uc3m.es (B.L.B.); vdiaz@ing.uc3m.es (V.D.); 2 Computer Science Department, Institute for Automotive Vehicle Safety (ISVA), Universidad Carlos III de Madrid, Avda. de la Universidad 30, 28911 Leganés, Madrid, Spain; lpgonzal@inf.uc3m.es (L.P.G.); jgarciag@inf.uc3m.es (J.G.G.)

**Keywords:** IoT, low-cost sensor, small single-board computer, Raspberry Pi, Intel Edison, low-cost devices, vehicle dynamics

## Abstract

Nowadays, the current vehicles are incorporating control systems in order to improve their stability and handling. These control systems need to know the vehicle dynamics through the variables (lateral acceleration, roll rate, roll angle, sideslip angle, etc.) that are obtained or estimated from sensors. For this goal, it is necessary to mount on vehicles not only low-cost sensors, but also low-cost embedded systems, which allow acquiring data from sensors and executing the developed algorithms to estimate and to control with novel higher speed computing. All these devices have to be integrated in an adequate architecture with enough performance in terms of accuracy, reliability and processing time. In this article, an architecture to carry out the estimation and control of vehicle dynamics has been developed. This architecture was designed considering the basic principles of IoT and integrates low-cost sensors and embedded hardware for orchestrating the experiments. A comparison of two different low-cost systems in terms of accuracy, acquisition time and reliability has been done. Both devices have been compared with the VBOX device from Racelogic, which has been used as the ground truth. The comparison has been made from tests carried out in a real vehicle. The lateral acceleration and roll rate have been analyzed in order to quantify the error of these devices.

## 1. Introduction

Given the high number of vehicle-crash victims, it has been established as a priority to reduce this figure in the transportation sector. For this reason, many of the recent research works are focused on including different control systems in commercial vehicles in order to improve their stability, comfort and handling [1,2,3]. These systems need to know in every moment the dynamics of the vehicle through variables such as longitudinal and lateral accelerations, yaw rate, roll rate, roll angle and sideslip angle, among others, when different maneuvers are performed, in order to actuate by means of the systems in the vehicle (brakes, steering, suspension) and, in this way, achieving a good behavior.

IMU sensors directly provide measurements of angular rates (yaw, roll and pitch rates) and accelerations (longitudinal, lateral and vertical accelerations) via rate gyroscopes and accelerometers. However, there are some difficulties in obtaining the vehicle angles (sideslip, roll and pitch angles) directly from sensors. A GPS dual-antenna system provides measurements of these angles, but this technique is very expensive. For this reason, this system is not mounted on commercial vehicles. Nowadays, many researches are focused on the design of observers in order to estimate these variables using the data provided by sensors that are installed on current vehicles, or by low-cost devices in order to solve this problem. In some studies, the vehicle roll angle is estimated by fusing the data of longitudinal acceleration, lateral acceleration, yaw rate and roll rate [4,5], through the fusion of lateral accelerations and roll rate [6], fusing the data obtained from an inertial angle sensor and a gyroscope [7], fusing the information obtained from a six-dimensional IMU [8] or fusing the information provided by a low-cost GPS with the data provided by a wheel speed sensor, steering angle sensor and a yaw rate sensor [9]. Regarding these works, the most common sensors used to estimate the vehicle dynamics are accelerometers and gyroscopes, which are integrated in an IMU sensor.

Concerning the estimation of vehicle sideslip angle, in [10], a sensor fusion of lateral acceleration, longitudinal velocity and yaw rate is carried out. In [11], the sideslip angle is estimated through the yaw rate. In [12,13], the estimation of sideslip angle is obtained through the lateral acceleration and yaw rate.

In [14], both the roll angle and sideslip angle are estimated using the measurements provide by sensors installed on commercial vehicles, such as the longitudinal velocity, the wheel speed, the steer angle, the lateral acceleration and yaw rate.

The observers’ design to estimate the vehicles angles is based on different techniques. These techniques are based on artificial intelligence [4,10], Kalman Filter [4,8,10], Bayesian Filters [9], sliding mode observer [6] and robust observers [5,11,13]. All these observers must work under real-time constraints.

Nowadays, some researches analyze and design embedded systems for vehicles’ applications [15,16,17]. With the objective of developing vehicle on-board systems, it is necessary that they have a small size, a small response time and enough accuracy. Another characteristic is that they must be low-cost in order to maintain the price of production vehicles. The use of small computers with a high processing capacity and a number of input/output interfaces allows incorporating into vehicles both estimators and controllers, increasing their safety. Previous research [18] analyzed the integration of low-cost sensors in vehicles. This research indicates that this kind of low-cost kit could present relevant bias and noise that need to be studied experimentally. Even more, this research provides recommendations for packaging these devices with plastic boxes. This research supports the need and opportunity for the research discussed in this article.

Small computers are used as data acquisition systems. Nowadays, the most popular small single-board computers are the Raspberry Pi and Intel Edison. They have a low price, flexibility and have high support from the creators and Internet community developing new libraries to give solutions for complex necessities. The use of these devices has been increasing in recent years.

The Intel Edison is meant to be a deeply-embedded IoT computing module. Although it is not a single-board computer, there are many studies that use it like an acquisition and processing system. Like Raspberry Pi, Intel Edison is very popular, and there are many resources to improve the capabilities of this processor. In [19], a prototype implementation based on the Intel Edison was used to verify an identity-based data aggregation protocol. In [20], real-time vital parameters on neonates were acquired using Intel Edison and IoT integration with biomedical devices. In [21], Intel Edison was used to develop a neurofeedback system to enable anyone with an attention deficit to practice regulating their brain to reach an attentive state of mind. In [22], Intel Edison was used to create a prototype to analyze and process geospatial data.

In [23], a Raspberry Pi was used to acquire the riding dynamics of a human-powered vehicle. In [24], a Raspberry Pi was used to develop an virtual laboratory, which allows the sensor and data fusion to be employed in autonomous, robotic and transportation systems. In [25], a low-cost contact angle goniometer/tensiometer was constructed based on a Raspberry Pi. In [26], a voltage regulation system was proposed for photovoltaic energy sources based on Raspberry Pi. The Raspberry Pi managed the data acquisition and controlled the system. In [27], the Raspberry Pi was used as a gateway for an efficient integration of wireless sensor networks with the Internet through IoT.

As can be seen, in previous works, these small computers are used for very specific works. In [28], the comparison of some of their inherent capabilities, such as connectivity and consumption, was performed under static conditions.

The novelty reflected in this work is the analysis of low-cost small computers and sensors under high dynamic conditions, by following specific best practices and considerations. The set of these devices is integrated with a specifically-designed IoT architecture oriented towards smart vehicles.

One of the main results of this research is the design of an architecture based on IoT, which integrates low-cost sensors and a small single-board computer to be mounted on a vehicle in order to carry out the estimation and control of vehicle dynamics, comparing the results with a high-end professional device. In this work, the low-cost sensor considered is an IMU due to it being the most common sensor used to estimate the vehicle dynamics. This research is focused on the comparison of two different low-cost systems (Raspberry Pi with IMU BNO0055 and Intel Edison with IMU LSM9DSO) in terms of accuracy, acquisition time and reliability. Both devices are going to be compared with the VBOX device from Racelogic, which will be used as the ground truth.

This article is organized as follows. In Section 2, the methodology is presented, including the experimental testbed design, experiments’ definitions, and the data gathering and analysis, the experimental results and the calculation of the RMS error are presented in Section 3. Finally, in Section 4, the discussion and conclusion of the results and the method are exposed.

## 2. Methodology

In this section, firstly, the design of the experimental testbed defined to achieve the research goals is presented; then, the experiments to gather the data required are specified; and finally, the data gathered to analyze low-cost sensor kits’ performance and reliability are presented, indicating the data analysis strategies for identifying relevant results and conclusions.

### 2.1. Experimental Testbed Design

The design of the experimental testbed considered for this research work is composed of three perspectives: hardware, software and communications.

**Figure 1:**
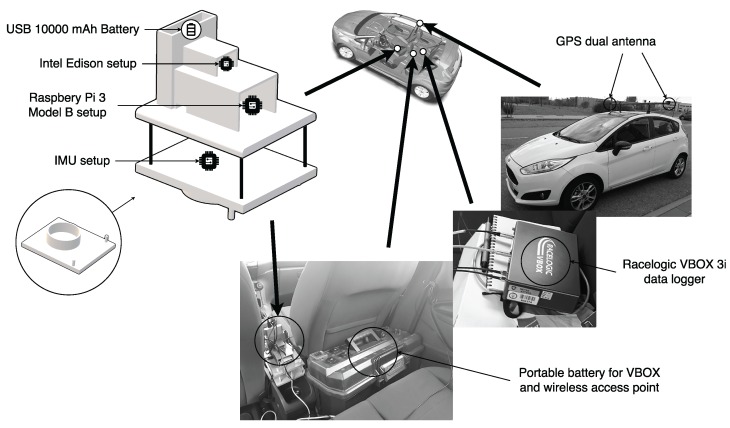
Test vehicle equipped with different low-cost systems, VBOX data logger, 3 IMU sensors and GPS dual-antenna.

**Figure 2:**
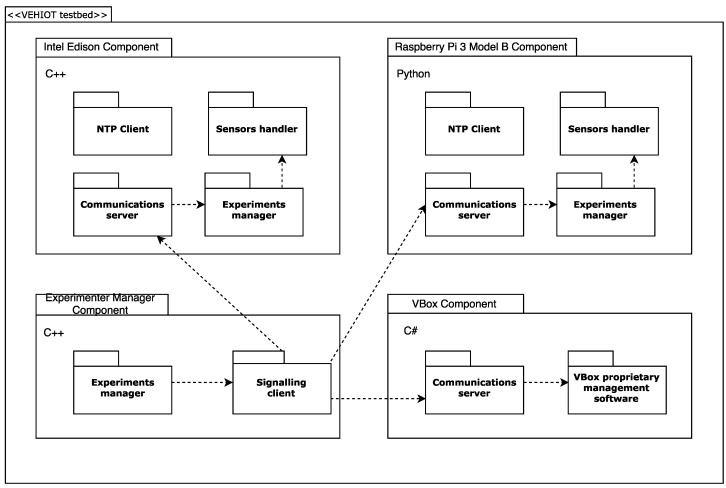
Testbed software design.

**Figure 3:**
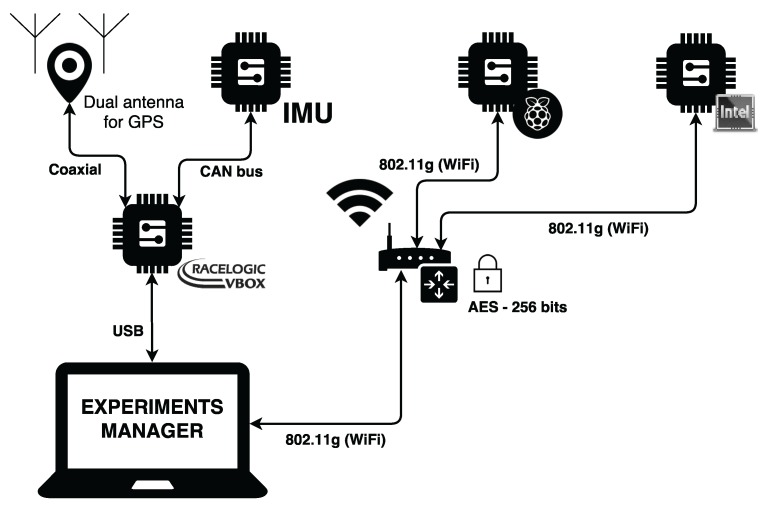
Testbed communications perspective.

- Hardware perspective:
  The experimental testbed is an Internet of Things (IoT) architecture embedded in a vehicle. Although the complete architecture is packaged in a product that can be integrated in any vehicle, the Ford Fiesta was used.
  To perform the comparative analysis properly, three kits of sensors were considered:
  – Reference or ground truth kit. This is composed of a VBOX 3i dual antenna data logger [29] having an IMU (Inertial Measurement Unit) connected and a dual antenna for incremental GPS from Racelogic. To accurately measure the roll rate, the two antennas form a 90-degree angle with respect to the traveling direction. For IoT kits’ synchronization and data gathering, the VBOX controller is connected to a laptop embedded in the vehicle. The actual specifications of the ground truth kit hardware elements are detailed in Table 1.
  – First low-cost sensor kit composed of a Raspberry Pi 3 Model B [30,31], low-cost Inertial Measurement Unit Shield [32]. The actual specifications of the Raspberry kit hardware elements are detailed in Table 2.
  – Second low-cost sensor kit composed of an Intel Edison System on Chip [33] linked to a SparkFun 9 Degrees of Freedommodule [34]. The actual specifications of the Intel kit hardware elements are detailed in Table 3.
  The IMU and the low-cost sensor kits were located in the gravity center of the vehicle considered in the testbed as is represented in Figure 1. These three kits were also interconnected using a WiFi router to manage the communications among them to synchronize the experiments and gather the information ready to be compared. According to [18], the accurate determination of IMU and controller position is essential for enhancing the precision of low-cost kits.
- Software perspective:
  A software architecture was designed to gather the data provided by the sensor kits in a synchronized way and to provide the datasets necessary to analyze the precision and performance of each kit considered for this research work. The main components of this architecture are shown in Figure 2).
  The Experiments Manager Componenthas the responsibility to synchronize the data-gathering of the kits taking part in the experiments. The specific classes included in this component are:
  1. Experiments Managerthat includes the user interface to let the researcher send a signal to the experimental kits for starting and finishing an experiment in a synchronized way.
  2. Communications Client that has the responsibility of managing the communication between the Experiments Manager and the Communications Server running in each experimental kit. The possible signals that can be sent from the Communications Client in the Experiments Manager and the Communications Servers in experimental kits are: 0, shutdown experimental kit; 1, keep running the experiment; 2, start the experiment; and 3, end the experiment and save the data in a file.
  3. Dataset Storage Manager that is in charge of taking the data coming from the kits and storing them in a CSV file. The information stored includes gyroscope and accelerator data gathered considering a sampling rate of 100 Hz.
  The Intel Edison Software component is in charge of gathering the information provided by gyroscope and accelerator sensors included in its hardware architecture. This component was implemented in C++. The specific classes included in this component are:
  1. The NTP Client is in charge of registering the actual date-time in the hardware controller of the experimental kit to ensure that all the kits in the testbed have the same date-time, supporting comparison during the data analysis stage in this research work.
  2. Communications Server that is in charge of receiving the signals from the Experiment Manager and sends the Experiment Launcher the order to start a new experiment. Moreover, this class sends the experiments’ ending order to the Experiment Stopper class to process the information gathered during the experiment.
  3. The Experiment Manager class is in charge of creating an empty data structure in RAM memory when it receives the start experiment signal from the communications server and sending the data structure having the data gathered during an specific experiment to the Experiments Manager for storage purposes when it receives the end experiment signal.
  4. The Sensors Handler class has the responsibility of registering data items from sensors attending to the pre-configured sampling rate (100 Hz for this research work). The data are obtained from the gyroscope and accelerometer drivers that were obtained from GitHub [35,36].
  The Raspberry Pi Software component has the same class structure as the Intel Edison Software Component, but it was developed in Python because the available drivers for low-cost IMU were only available in this language.
  The VBOX Component is in charge of gathering the information provided from the Racelogic IMU sensor and GPS dual antenna data.
- Communications perspective:
  In order to provide homogeneity in communication among the different items that compose the solution and ensure synchronization when performing the different tests, a communications architecture has been defined.This architecture is shown in Figure 3.
  Given the nature of Racelogic VBOX devices, they need to be physically connected via a cable to the experiments managerand among themselves. However, both Raspberry Pi 3 and Intel Edison come with wireless communication interfaces that ease the connectivity among components and allow one to locate them in virtually any place of the vehicle without worrying about setting up specific data cables. Even more, the sensors used by these low-cost platforms are straightforwardly attached to the development boards by using the GPIO ports. By means of a wireless (802.11 g) access point, they can be connected to the experiments manager, which signals to them their operation mode via a TCP socket connection.

### 2.2. Experiments Definition

The hypotheses to evaluate during this research work are the following:

- H1: The precision of low-cost sensor kits is similar to the precision provided by expensive experimental kits (i.e., VBOX-based kits).
- H2: The performance and reliability of the low-cost sensor kits (i.e., Raspberry Pi and Edison Kits) is similar to the performance and reliability provided by expensive experimental kits (i.e., VBOX-based kits).

To evaluate the previous hypothesis, eight controlled experiments were executed (see Table 4. The experiments considered typical maneuvers such as J-turn and lane change maneuvers. These maneuvers are the most common used to test the vehicle’s behavior. Furthermore, a long test simulating a general execution has been done. In the experiments carried out, the lateral acceleration and the roll rate are the variables that have suffered higher variation, and for this reason, these variables are considered in order to analyze the accuracy of different devices.

**Figure 4:**
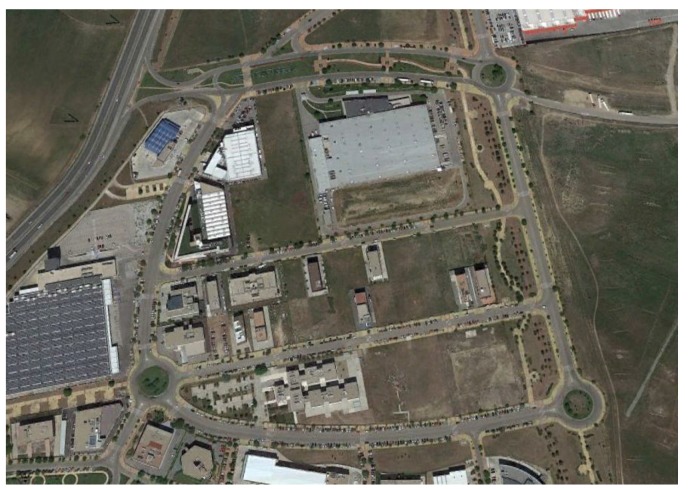
Experiments’ context (Map scale 1:7800 cm).

The experimental tests have been carried out in Leganes (Madrid, Spain) on a commercial vehicle and a Ford Fiesta, as can be seen in Figure 4 in a period were the setting had no traffic restrictions interfering with the appropriate execution of the considered experiments.

### 2.3. Data Gathering and Analysis

**Figure 5:**
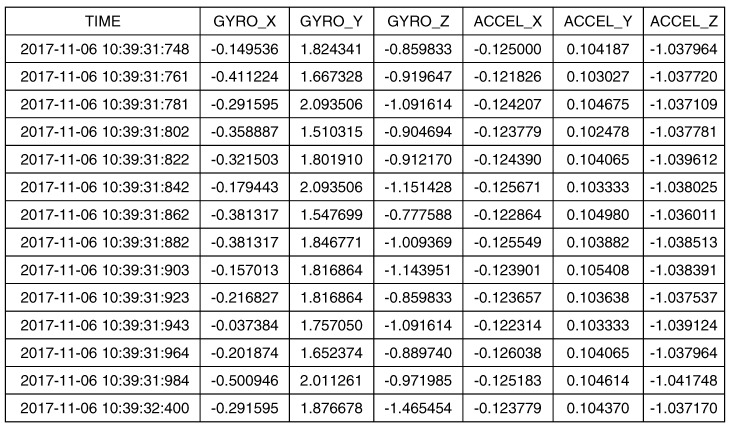
Example of the file including data registered during the experiments’ execution.

The data obtained for the sensors considered for the previously-defined experiments were: For each experiment, the controller of each kit stored a CSV formatted file identifying the experiment and its execution date and time. The variables considered were the lateral acceleration measured by the accelerometer and the roll rate measured by the gyroscope included in each kit for the three axes. The measures were gathered according to the sampling rate stated for the experiments, which was 100 Hz. Figure 5 presents an example of the data gathered in each experiment.

To determine the accuracy of the values obtained from low-cost kits, the root mean square (RMS) error was calculated. The maximum error comparing each point has also been calculated. This comparisons has been done using Racelogic VBOX as the ground truth.

Section 3 presents the results obtained in the different tests.

### 2.4. Threats to Validity

In order to analyze the validity of the results obtained in this experimental work, several threats were considered prior to the experiments’ execution:

- Internal validity is the extent to which a causal conclusion based on the experiments defined is appropriate avoiding the introduction of systematic errors in the data used to determine the results and conclusions.
- External validity is the extent to which the results of a study can be generalized to other situations.

(A) Internal validity: In this research, the only factor contributing to internal validity is related to the specific sensors used to configure each low-cost kit and the software components implemented to manage the required data:

- The first threat was mitigated using two different kits for each type in order to prevent errors produced by sensors providing incorrect values. Even more, all the kits considered were tested in a static environment configuring the corresponding calibrations to assure that specific sensors included were providing appropriate data.
- Regarding software components, the possible threats were mitigated implementing an exhaustive unit testing process to ensure that each functionality properly processes the received values and the synchronization among devices is correctly implemented.
- To verify the validity of the results, three similar tests for J-turn and double lane change maneuvers have been carried out.

(B) External validity: In the scope of this research, the factors that influence the external validity are related to the replication of this experiment. These replications must consider several relevant factors: sensors and controllers included in the experimental kits, their location in the vehicle and road conditions:

- Regarding sensors and controllers, the possible threats were mitigated using sensors and controllers available on the market having regular features [37,38,39]. In this sense, the conclusions obtained are valid for the low-cost sensors currently available on the market, and as the technology is always improving the prior conditions, the conclusions can be used for future low-cost sensors.
- Regarding vehicle conditions, the threats were related to the appropriate location of experimental kits in order to ensure equal conditions among them. This threat was mitigated creating a box to put the three sensor kits in a 3D printed box that was located in the vehicle gravity center. These issues are important to replicate this experiment properly in other vehicles. In this research, as the experiments have been carried out with a real vehicle, it has been difficult to maintain the same exact driving conditions related to steering wheel angle and velocity. In spite of this problem, similar tests for typical maneuvers have been carried out.
- Regarding road conditions and experiments execution, the threats were related to the representativeness of the scenarios considered. This threat was mitigated considering a road without relevant slope variations and including different experiments with different types of directions, constant and variable speed.

## 3. Experimental Results

As is indicated in Section 2, a Ford Fiesta was used for this research (Figure 1). For the experimental tests, a total of eight driving maneuvers was carried out. Table 5 shows a summary of the successful tests. For both VBOX and Intel Edison, the percentage of successful tests is 100%. However, the percentage of successful tests for Raspberry Pi is 37.5%. This result together with the connectivity problems suffered previously in the performance of tests indicates a low reliability of Raspberry Pi compared with the other devices. In Table 6, the vehicle speed for the three successful tests in three devices is shown.

### 3.1. Test 1: Roundabout

This first test is carried out with the vehicle taking a roundabout with a radius of 22 m (see Figure 7) at a constant speed on dry pavement. The car takes the roundabout with a velocity of 31 km/h. Figure 8 and Figure 9 show the lateral acceleration and roll rate data, respectively, obtained from the lateral accelerometers and roll rate sensors contained in IMUs installed on Raspberry Pi (blue points) and Intel Edison (green). In order to prove the accuracy of these sensors, they have been compared with the data obtained from the IMU of VBOX, which is considered as the ground truth. It can been seen that the behaviors of the sensors in the three different devices are very similar.

To quantify the accuracy of the sensor, the norm, RMS (root mean square) and maximum errors have been calculated. The norm error as a function of time is calculated as follows [5]:

(1) $$E_{t}=\frac{\varepsilon_{t}}{\sigma_{t}}\cdot 100,$$

where:

(2) $$\begin{array}{c} \varepsilon_{t}^{2}=\int_{0}^{T}{\left(\phi_{GT}-\phi_{lc}\right)}^{2}dt\\ \sigma_{t}^{2}=\int_{0}^{T}{\left(\phi_{GT}-\mu_{GT}\right)}^{2}dt \end{array}$$

$\phi_{GT}$ represents the ground truth data, $\phi_{lc}$ represents the low-cost sensor data and $\mu_{GT}$ is the mean value of the ground truth data obtained during the period T .

In Table 7, the error values are given. To verify the validity of the results, three similar tests for the J-turn maneuver have been carried out. To quantify the dispersion of data values, the standard deviation has been included for the RMS error (see Table 7). The results show that the errors are higher for the Intel Edison than the Raspberry Pi. Concerning the norm and RMS errors, the difference is about 1%, 0.01 g’s and 3%, 0.02${}^{\circ }$/s for lateral acceleration and roll rate, respectively. For maximum errors, the difference between them is higher (about 0.2 g’s and 6${}^{\circ }$/s, for lateral acceleration and roll rate, respectively). This is due to the Intel Edison being more sensitive to noise. In Figure 8 and Figure 9, a high scattering for the Intel Edison IMU is observed.

### 3.2. Test 2: Double Lane Change

This second test is carried out with the vehicle doing a slalom maneuver at 79 km/h on dry pavement as shown in Figure 10. In this kind of test, the lateral acceleration varies very fast, so the sampling frequency of the devices and sensors can be checked. Figure 11 and Figure 12 show the lateral acceleration and roll rate data, respectively, obtained from the lateral accelerometers and roll rate sensors contained in IMUs installed on Raspberry Pi (blue points) and Intel Edison (green). In order to prove the accuracy of these sensors, they have been compared with the data obtained from the IMU of VBOX, which is considered as the ground truth. It can been seen that the behavior of the sensors in the three different devices is very similar. Furthermore, it can be seen that the two devices are able to sample the signal sufficiently to show no differences with the Racelogic VBOX device.

To quantify the accuracy of the sensor, the norm, RMS and maximum errors have been calculated. In Table 8, the error values are given. To verify the validity of the results, three similar tests for the double lane change maneuver have been carried out. The standard deviation for the RMS error is shown in Table 8. Results show that the errors are higher for Intel Edison than Raspberry Pi. Concerning the norm and RMS errors, the difference is about 20%, 0.04 g’s and 65%, 2${}^{\circ }$/s for lateral acceleration and roll rate, respectively. For maximum errors, the difference between them is higher (about 0.5 g’s and 57${}^{\circ }$/s, for lateral acceleration and roll rate, respectively). This is due to the Intel Edison being more sensitive to noise. In Figure 11 and Figure 12, a high scattering for Intel Edison IMU is observed; it also can be seen that the maximum error for roll rate on Intel Edison is 66.0722${}^{\circ }$/s. This error is due to the noise that causes atypical data, as can be see in Figure 13.

### 3.3. Test 3: General Circulation

Compared to the tests described above, in this case, there are not only severe maneuvers, low and medium speed circulation and smooth movements are performed. At the end of the trial, two roundabouts (J-turn maneuver) and a Lane Change (LC maneuver) at a speed suitable for the testing environment have been made. It should be considered that in this test, low speed movements and high speed severe movements have been made.

**Figure 6:**
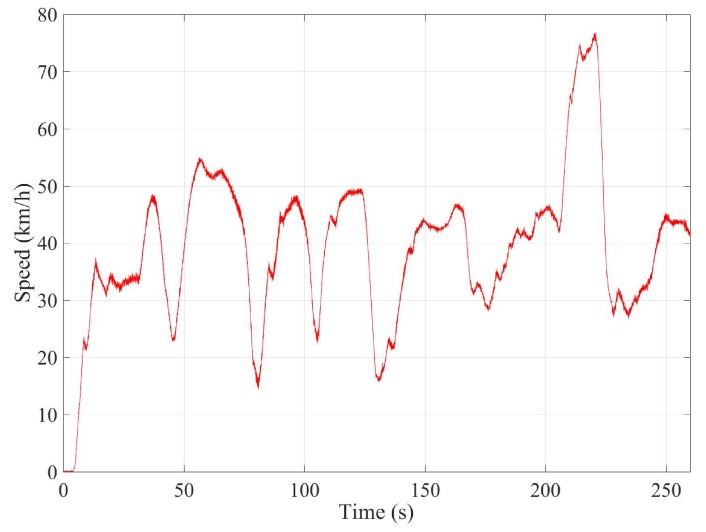
Test 3: Vehicle speed profile.

**Figure 7:**
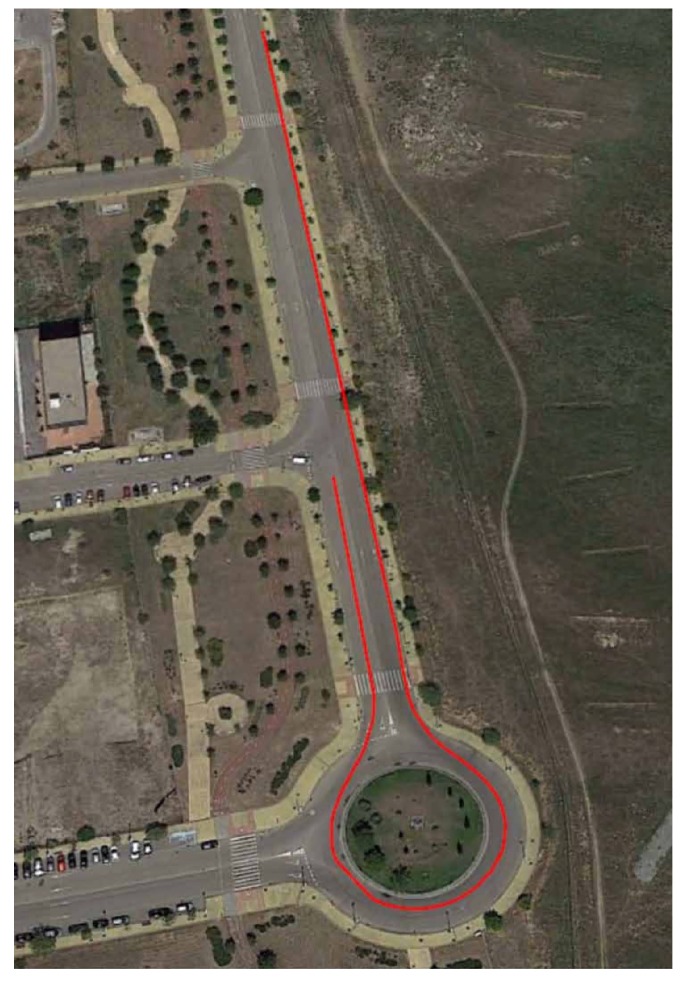
Test 1: Map and vehicle trajectory (Map scale 1:2100 cm).

**Figure 8:**
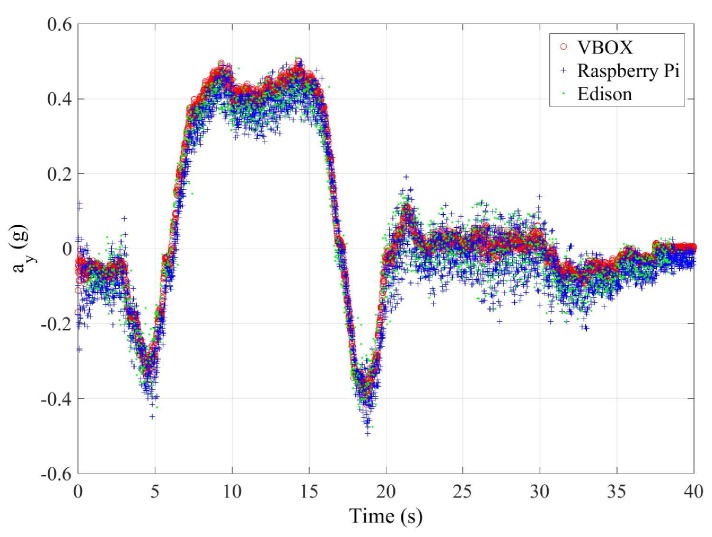
Test 1: Lateral acceleration obtained from the IMU of VBOX (red points), from the IMU of Raspberry pi (blue points) and from the IMU from Intel Edison (green points).

**Figure 9:**
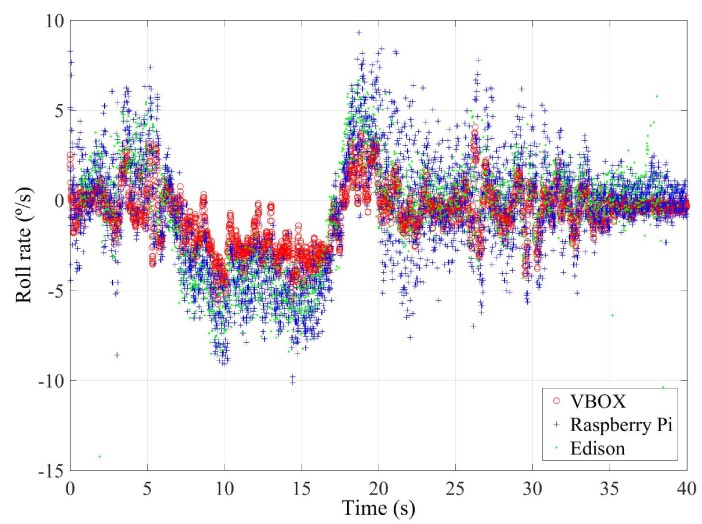
Test 1: Roll rate obtained from IMU of VBOX (red points), from IMU of Raspberry pi (blue points) and from IMU from Intel Edison (green points).

**Figure 10:**
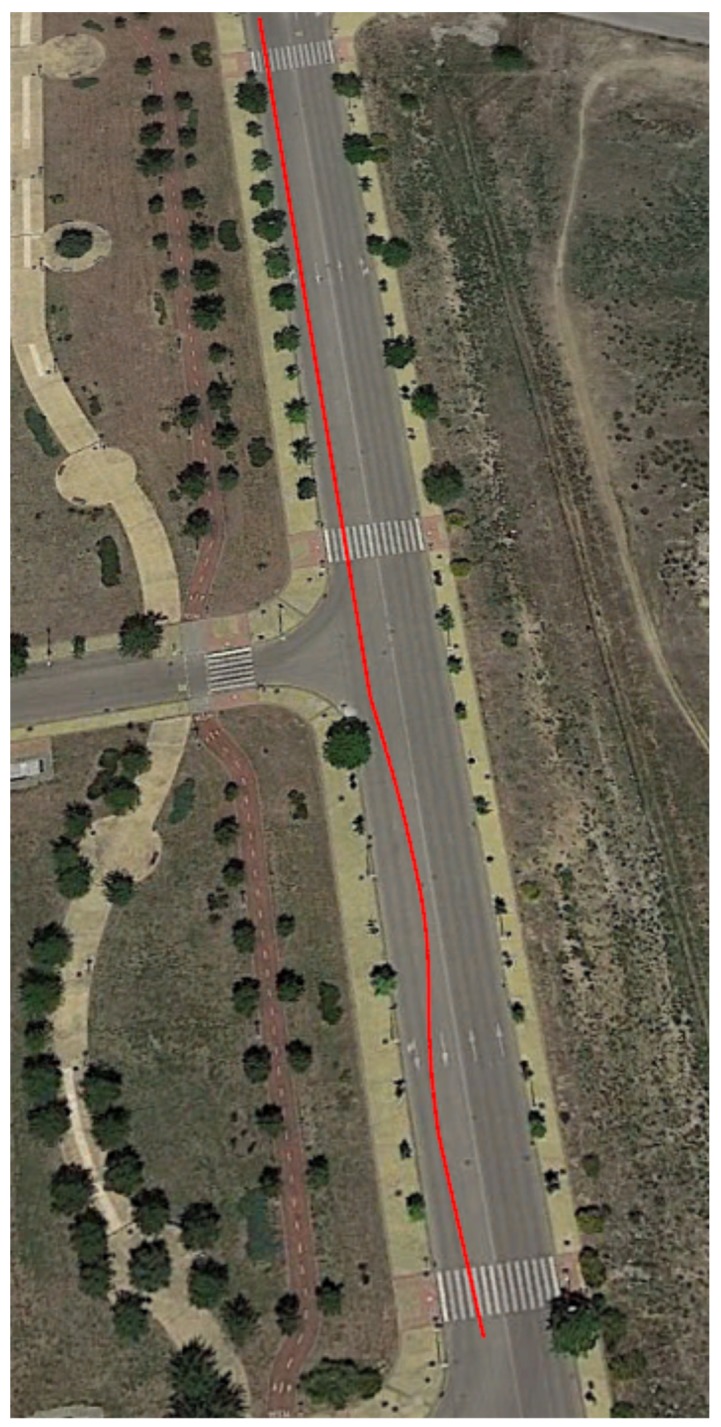
Test 2: Map and vehicle trajectory (Map scale 1:2100 cm).

**Figure 11:**
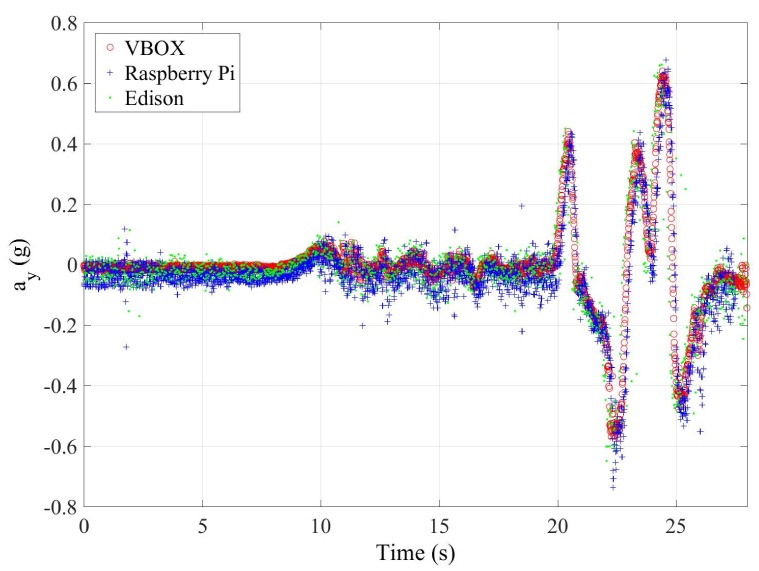
Test 2: Lateral acceleration obtained from the IMU of VBOX (red points), from the IMU of Raspberry pi (blue points) and from the IMU from Intel Edison (green points).

**Figure 12:**
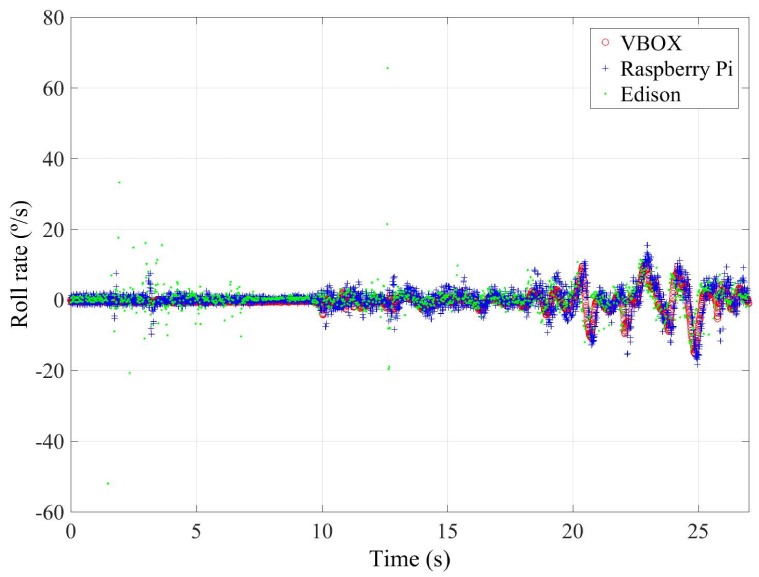
Test 2: Roll rate obtained from the IMU of VBOX (red points), from the IMU of Raspberry pi (blue points) and from the IMU from Intel Edison (green points).

**Figure 13:**
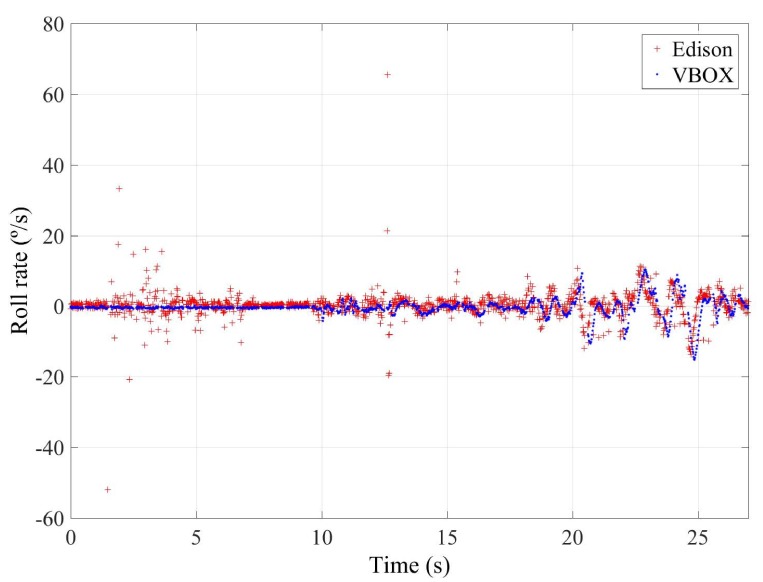
Test 2: Roll rate obtained from the IMU of VBOX (blue points) and from the IMU from Intel Edison (red points).

**Figure 14:**
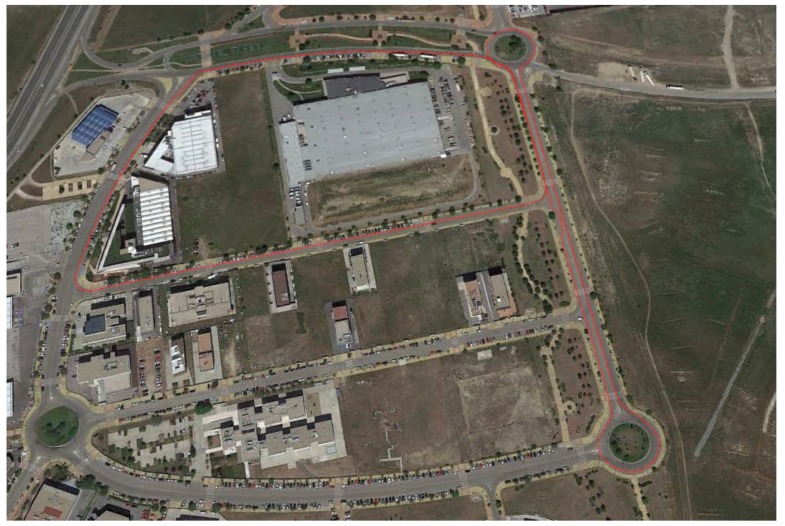
Test 3: Map and vehicle trajectory (Map scale 1:5036 cm).

**Figure 15:**
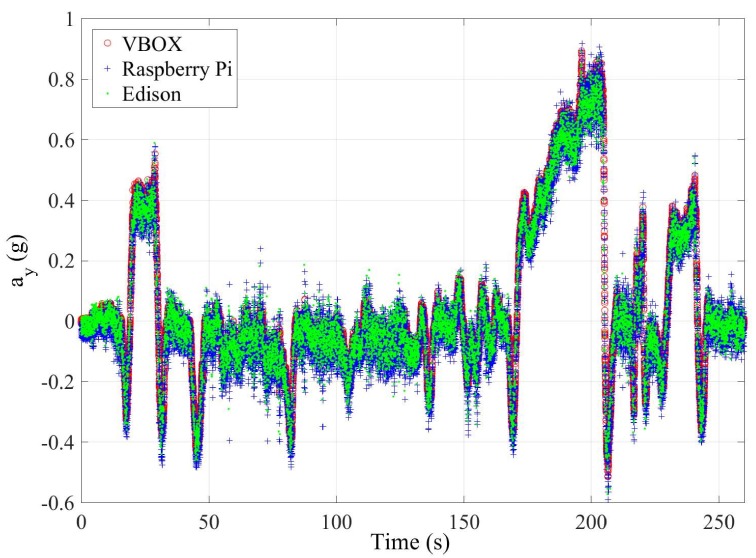
Test 3: Lateral acceleration obtained from the IMU of VBOX (red points), from the IMU of Raspberry pi (blue points) and from the IMU from Intel Edison (green points).

**Figure 16:**
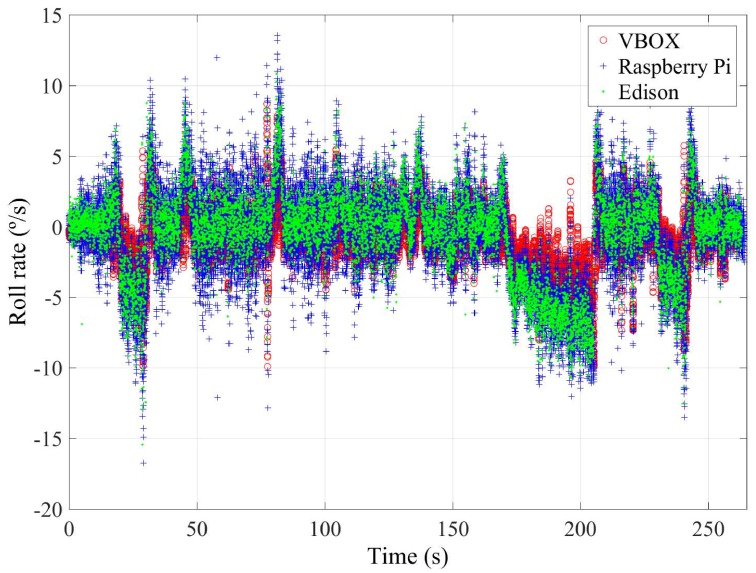
Test 3: Roll rate obtained from the IMU of VBOX (red points), from the IMU of Raspberry pi (blue points) and from the IMU from Intel Edison (green points).

This last test is carried out on the route shown in Figure 14. This test simulates a normal circulation behavior. Several curves were taken, and the vehicle was at the most appropriate speed for the road and the situation. The speed range on this test was from 15 km/h–75 km/h (see Figure 6). Figure 15 and Figure 16 show the lateral acceleration and roll rate data, respectively, obtained from the lateral accelerometers and roll rate sensors contained in IMUs installed on Raspberry Pi (blue points) and Intel Edison (green). In order to prove the accuracy of these sensors, they have been compared with the data obtained from the IMU of VBOX, which is considered as the ground truth. It can been seen that the behaviors of the sensors in the three different devices are very similar.

To quantify the accuracy of sensor, the norm, RMS and maximum errors have been calculated. In Table 9, it can be observed that the errors are higher for Intel Edison than the Raspberry Pi. Concerning the norm and RMS errors, the difference is about 2%, 0.007 g’s and 18%, 0.35${}^{\circ }$/s for lateral acceleration and roll rate, respectively. For maximum errors, the difference between them is higher (about 0.3 g’s and 5${}^{\circ }$/s, for lateral acceleration and roll rate, respectively). This is due to the Intel Edison being more sensitive to noise. In Figure 15 and Figure 16, a high scattering for the Intel Edison IMU is observed. However, the error on this test is lower than the other two. The reason is that the highest value for lateral acceleration was reached, so the influence of noise decreases compared to the measured value.

## 4. Discussion and Conclusions

The results can be used to design, implement and test an efficient, versatile and scalable low-cost hardware/software architectures able to be integrated on commercial vehicles. Even more, by using this sensor fusion approach with enhanced semantics, it may be possible to perform real-time estimation and control for more secure driving.

The following discussion is focused on the precision and performance similarity among precision and performance in both experimental low-cost and ground truth kits.

### 4.1. Precision

The obtained data show that low-cost sensors are more prone to noise. One reason is that their measurement range is higher than the VBOX ones. In case a noise reduction for the low-cost kit captured data were required, with the consequent approximation to the VBOX results, it may be possible to integrate filters via software, as the low-cost controllers can assume such a computational cost reducing the underutilization of their multiprocessing capabilities. Despite the noise influence, the average RMS error in BNO055 is 0.05 g’s (0.4905 m/s${}^{2}$) for lateral acceleration and 2${}^{\circ }$/s (0.0349 rad/s) for roll rate, and the average RMS error in LSM9DSO is 0.07 g’s (0.6867 m/s${}^{2}$) for lateral acceleration and 3${}^{\circ }$/s (0.0523 rad/s) for roll rate.

Furthermore, it has been found that low-cost accelerometers provide better precision than gyroscopes. A feasible justification is that the relative position among sensors guarantees the same acceleration measurement for the three devices. BNO055 has been proven to capture better results than LSM9DSO (with an approximate error of 0.02 g’s (0.1962 m/s${}^{2}$) and 1${}^{\circ }$/s (0.01745 rad/s) less for BNO055). Notice that BNO055 is more expensive than the later one. As future work, it is planned to integrate BNO055 in the Intel Edison setup via the Sparkfun GPIO expansion board and also to integrate software estimators and controllers and analyze their performance in small computers using the information captured by the low-cost sensors.

### 4.2. Reliability

After concluding the experiments, a problem was identified regarding Raspberry Pi 3. A significant number of tests was not valid. It was concluded that most of these failed tests resulted from the lack of true parallelism in the Python main stack (to get true parallelism, it is necessary to call low level C routines [40]). Even more, BNO055 was attached to Raspberry Pi 3 in a circuit board without industrial-grade soldering connections, which can induct noise and additional impedances in the circuit.

Finally, VBOX can provide a sustained 100-Hz capture rate, as accelerometer and gyroscope sensors for the low-cost versions; however, given that the magnetometer is embedded and also initialized (but not used) with the previous two, the sampling rate decreases to 50 Hz. In any case, experiments determined that 50 Hz is enough sampling frequency to perform reliable experiments for the given case [41].

### 4.3. Factors to Replicate and Evaluate Experiments Using Low-Cost Sensors

Some important aspects have been determined in order to properly replicate and evaluate the experiments of the study:

- Vehicle: The chosen vehicle perfectly fulfills the established requirements. This work is focused on commercial vehicles, so the tests must be carried out in one vehicle of these characteristics, in order to be exposed to the same control systems integrated in the vehicle (ESC, ABS, etc.).
- Track: To take a proper measurement of sensors’ reliability, the ideal scenario consists of having a test track without great camber and slope variations, as they can interfere with the collected data because of the lack of capacity to directly measure them.
- Hardware: To obtain accurate values, it is necessary to properly fix the setup position inside of the vehicle. It must be as close as possible to the vehicle’s center of mass. In addition, the relative position between the devices should be as small as possible, and the sensors must be aligned on the axis according to the characteristic to be measured (acceleration and angular velocity).
  Regarding hardware, as indicated in [18], effective mechanical and software calibration of low-cost IMU devices is important to reduce the bias and noise in this kind of experimental kit. The use of VBox equipment was essential to complete this step by appropriately analyzing the reliability of low-cost sensor kits. Moreover, the integration at the hardware level assures higher reliability levels, avoiding kits without industrial-grade soldering connections.
- Software: Regarding synchronization and capture software components, the preferred environment consists of using a well-proven and efficient programming language that allows one to take direct advantage of parallel hardware features, such as C or C++, in conjunction with efficient compilers that outcome lightweight and optimized binaries. This increases the results’ reliability and the experiments’ performance. Furthermore, it is important to define a unified test kit that allows one to verify, prior to starting the experiments, that all the elements in the setup are behaving properly.

## Figures and Tables

**Table 1:** Technical specifications of hardware elements included in the VBOX kit (ground truth).

VBOX 3i Data Logger Plus GPS Dual Antenna				IMU (RLVBIMU04)	
**Latency**	8.5 ± 1.5 ms	**Memory**	Compact Flash: Type I	**Angular rate range**	±150${}^{\circ }$/s
**Sampling rate**	100 Hz	**Velocity accuracy**	0.1 km/h	**Acceleration range**	±1.7 g
**Velocity range**	from 1000 mph to 0.1 Km/h	**Power consumption**	Max. 5.5 Watts	**Angular rate resolution**	0.01${}^{\circ }$/s
**Weight**	C	**Size**	170 × 121 × 41 mm	**Acceleration resolution**	0.01 g
**Price**		>13,000 €		**Price**	>3000 €

**Table 2:** Technical specifications of hardware elements included in the Raspberry Pi kit.

Raspberry Pi Controller (3 Model B)		IMU (BNO055)	
**RAM**	1 GB	**Angular rate range**	From ±125${}^{\circ }$/s–±2000${}^{\circ }$/s
**CPU**	4*x* ARM Cortex-A53, 1.2 GHz	**Acceleration range**	From ±2 g–±16 g
**GPIO**	40 pins on 0.1” headers	**Angular rate resolution**	16 bits (From 0.003${}^{\circ }$/s for ±125${}^{\circ }$/s to 0.06${}^{\circ }$/s for ±2000${}^{\circ }$/s)
**Power consumption**	5 V @ < 1.5 W–6 W^*o*^	**Acceleration resolution**	14 bits (From 0.0002 g for ±2 g to 0.002 g for ±16 g)
**Dimensions**	85.60 × 56.5 mm		
**Price**	33.70 €	**Price**	29.50 €

**Table 3:** Technical specifications of hardware elements included in the Intel Edison kit.

Intel Edison Controller		IMU (LSM9DSO)	
**RAM**	1 GB	**Angular rate range**	From ±245${}^{\circ }$/s– ±2000${}^{\circ }$/s
**CPU**	4*x* Intel Atom Tangier x86 dual core processor +Intel Quark core	**Acceleration range**	From ±2 g–±16 g
**GPIO**	70-pin Hirose. 4 mm	**Angular rate resolution**	16 bits (From 0.007${}^{\circ }$/s for ±245${}^{\circ }$/s to 0.06${}^{\circ }$/s for ±2000${}^{\circ }$/s)
**Power consumption**	3.3 V @ < 1 W	**Acceleration resolution**	14 bits (From 0.0002 g for ±2 g to 0.002 g for ±16 g)
**Dimensions**	35.5 × 25 mm		
**Price**	42.00 €	**Price**	13.50 €

**Table 4:** Experiments proposed.

ID	Description	Purpose	Variables to Observe
1	A vehicle taking two roundabouts with a radius of around 20 m at a constant speed of approximately 30 km/h.	(1) Evaluation of measures accuracy	Lateral acceleration and roll rate
2	A vehicle doing a lane change at approximately 20 km/h.	(1) Evaluation of measures accuracy	Lateral acceleration and roll rate
3	A vehicle taking several roundabouts with a radius of around 20 m at aconstant speed of approximately 35 km/h.	(1) Evaluation of measures accuracy	Lateral acceleration and roll rate
4	A vehicle taking several roundabouts with a radius of around 20 m at a constant speed of approximately 45 km/h.	(1) Evaluation of measures accuracy	Lateral acceleration and roll rate
5	A vehicle doing a lane change at approximately 60 km/h.	(1) Evaluation of measures accuracy	Lateral acceleration and roll rate
6	A vehicle taking a single roundabout with a radius of around 20 m at aconstant speed of approximately 30 km/h.	(1) Evaluation of measures accuracy	Lateral acceleration and roll rate
7	A vehicle doing a lane change at approximately 80 km/h.	(1) Evaluation of measures precision (2) Performance and reliability evaluation, specifically sampling frequency of the devices and sensors	Lateral acceleration and roll rate
8	A vehicle simulates a normal circulation behavior. Several curves were taken, and the vehicle was at the most appropriate speed for the road and the situation.	(1) Evaluation of measures precision (2) Performance and reliability evaluation, specifically sampling frequency of the devices and sensors	Lateral acceleration and roll rate

**Table 5:** Results of reliability.

	VBOX	Raspberry Pi	Intel Edison
Total tests	8	8	8
Successful tests	8	3	8
% of reliability	100	37.5	100

**Table 6:** Vehicle speed for tests.

Maneuver	Speed (km/h)
Test 1: J-turn	31
Test 2: Lane change	79
Test 3: Normal driving	Variable (see Figure 6)

**Table 7:** Test 1: Errors of lateral acceleration and roll rate data for the accelerometers and gyroscopes mounted on Raspberry Pi and Intel Edison compared with the IMU from VBOX (ground truth).

	**Lateral Acceleration**				
	**Norm Error**	**RMS Error**		**Maximum Error**	
	**(%)**	**(g’s)**	**(m/s${}^{2}$)**	**(g’s)**	**(m/s${}^{2}$)**
Raspberry Pi	24.27	0.0541 ± 0.0041	0.5305 ± 0.04	0.2063	2.0238
Intel Edison	25.08	0.0692 ± 0.0072	0.6788 ± 0.0706	0.3844	3.7709
	**Roll Rate**				
	**Norm Error**	**RMS Error**		**Maximum Error**	
	**(%)**	**(${}^{\circ }$/s)**	**(rad/s)**	**(${}^{\circ }$/s)**	**(rad/s)**
Raspberry Pi	143.15	2.2737 ± 0.1555	0.039 ± 0.0027	8.3404	0.1455
Intel Edison	146.43	2.3093 ± 0.4153	0.04029 ± 0.0072	14.3173	0.2498

**Table 8:** Test 2: Errors of lateral acceleration and roll rate data for the accelerometers and gyroscopes mounted on Raspberry Pi and Intel Edison compared with the IMU from VBOX (ground truth).

	**Lateral Acceleration**				
	**Norm Error**	**RMS Error**		**Maximum Error**	
	(**%)**	**(g’s)**	**(m/s**${}^{2}$**)**	**(g’s)**	**(m/s**${}^{2}$**)**
Raspberry Pi	46.48	0.0447 ± 0.0097	0.4385 ± 0.0951	0.2655	2.6045
Intel Edison	68.44	0.0842 ± 0.0175	0.8260 ± 0.1716	0.7863	7.7136
	**Roll Rate**				
	**Norm Error**	**RMS Error**		**Maximum Error**	
	**(%)**	**(**${}^{\circ }$**/s)**	**(rad/s)**	**(**${}^{\circ }$**/s)**	**(rad/s)**
Raspberry Pi	98.92	1.7007 ± 0.5142	0.0296 ± 0.0089	9.2531	0.1614
Intel Edison	163.66	3.8715 ± 1.1463	0.0675 ± 0.02	66.0722	1.1529

**Table 9:** Test 3: Errors of lateral acceleration and roll rate data for the accelerometers and gyroscopes mounted on Raspberry Pi and Intel Edison compared with the IMU from VBOX (ground truth).

	**Lateral Acceleration**				
	**Norm Error**	**RMS Error**		**Maximum Error**	
	**(%)**	**(g’s)**	**(m/s**${}^{2}$**)**	**(g’s)**	**(m/s**${}^{2}$**)**
Raspberry Pi	21.4	0.0525	0.5150	0.3491	3.4246
Intel Edison	23.81	0.0591	0.5798	0.6142	6.0253
	**Roll Rate**				
	**Norm Error**	**RMS Error**		**Maximum Error**	
	**(%)**	**(**${}^{\circ }$**/s)**	**(rad/s)**	**(**${}^{\circ }$**/s)**	**(rad/s)**
Raspberry Pi	113.6	2.0583	0.0359	11.8485	0.2067
Intel Edison	131.97	2.4074	0.0420	16.0820	0.2806

## Abbreviations

The following abbreviations are used in this manuscript:

GPS	Global Positioning System
IMU	Inertial Measurement Unit
IoT	Internet Of Things
RMS	Root Mean Square
